# Pilonidal sinus disease: Review of current practice and prospects for endoscopic treatment

**DOI:** 10.1016/j.amsu.2020.07.050

**Published:** 2020-08-01

**Authors:** Fahad Mahmood, Anwar Hussain, Akinfemi Akingboye

**Affiliations:** a Department of General and Colorectal Surgery, Russells Hall Hospital, Pensnett Rd, Dudley, West Midlands, DY1 2HQ, UK; b Department of General and Colorectal Surgery, Leighton Hospital, Middlewich Road, Crewe, Cheshire, CW1 4QJ, UK

**Keywords:** Pilonidal sinus disease, Endoscopic pilonidal sinus treatment, Video-assisted ablation of pilonidal sinus, Excision of pilonidal sinus, Karydakis procedure, Bascoms procedure

## Abstract

Pilonidal sinus disease is chronic acquired condition leading to significant morbidity and associated healthcare costs. Several techniques have been described to manage this condition with no treatment gaining universal acceptance. With the shift towards minimally invasive surgery, Video Assisted-Ablation of Pilonidal Sinus (VAAPS) and Endoscopic Pilonidal Sinus Treatment (EPiST) have gained prominence. The aim of this review is to analyse current treatment modalities and the evidence for endoscopic pilonidal sinus surgery.

Reported surgical techniques range from wide excision with or without primary closure to various flap closures. These aim to eliminate the underlying causes driven by natal cleft hair and reducing recurrence. However, long term (≥5 years) recurrence rates range between 10 and 30% with significant complication rates. Trials with endoscopic treatment which have shown comparable short-term results to established treatments with reduced morbidity. However, the potential higher cost, learning curve, patient selection criteria and need for long term outcomes from randomised trials limit widespread application of this promising method.

Endoscopic treatment of pilonidal sinus disease therefore provides a minimally invasive alternative to traditional surgical methods with the potential to reduce morbidity. However long-term outcomes data from further prospective randomised trials is needed to establish its efficacy compared to traditional surgical methods.

## Highlights

• Multiple treatment modalities for pilonidal sinus disease exist with no universally accepted mode of treatment.
• Pilonidal sinus disease recurrence is high even after treatment.
• Endoscopic treatment has comparable short-term results to established treatments with reduced morbidity.
• Video Assisted-Ablation of Pilonidal Sinus and Endoscopic Pilonidal Sinus Treatment are emerging treatment modalities.

## 1 Historical background & epidemiology

Pilonidal disease was originally described by Herbert Mayo in 1833 as a congenital condition with the term ‘pilonidal’, derived from the Latin ‘nest of hairs’, being coined by Richard Hodges in 1880 [1]. Diagnosis was through identifying a characteristic epithelial track (the sinus) located in the skin of the natal cleft. During the Second World War the condition was common in jeep drivers, hence the term ‘jeep disease’ [2,3]. Moreover, a similar condition was identified in the interdigital clefts of clefts of barbers caused by hair entering moist, damaged skin [4]. Current understanding acknowledges pilonidal disease as an acquired chronic infection of the natal cleft skin and subcutaneous tissue that manifests acutely or with intermittent symptoms over several years.

Pilonidal disease has a reported incidence of 26 per 100,000 in the US [5] and 48 per 100,000 in Germany [6]. The condition is more common in Caucasians due to hair characteristics and growth patterns [5,7], typically affecting the teenage to young adult population up to the 3rd decade. The mean age of presentation is 21 years in men and 19 years in women [8]. Furthermore, the prevalence amongst men is two to three times that of women [9,10]. Therefore, pilonidal disease represents a significant disease burden, affecting people in their most productive years with huge socioeconomic implications. In its most severe form, pilonidal disease can be severely debilitating, causing daily discomfort and limiting activity.

A wide range of treatment options have evolved with rates of recurrence and morbidity from traditional surgical approaches unacceptably high. This review evaluates current and future treatment modalities considering the evolving understanding of disease pathophysiology. These highlight the need to critically re-evaluate the surgical treatment of pilonidal disease and embrace newer treatment modalities. Moreover, non-surgical treatment for uncomplicated pilonidal disease is gaining popularity where efficacy needs to be evaluated in light of current evidence.

## 2 Pathophysiology

Pilonidal disease was thought be of congenital origin, but increasing evidence indicates an acquired aetiology [11,12]. Firstly, occupation plays a major role with reports of occurrences between the fingers of sheep shearers, dog groomers, and barbers [4]. Further risk factors include, a sedentary lifestyle, positive family history, obesity, hirsute body habitus, local irritation or trauma [5,11]. Secondly, blocked hair follicles can lead to enlargement and rupture of the pilosebaceous glands with either abscess formation or a chronically discharging sinus [13]. In addition, Bascom postulated pilonidal disease as originating from a stretched midline hair follicle of the epidermal skin layer, analogous to an epidermal inclusion microcyst, thereby advising against resecting deep tissue during surgery [7]. However, Karydakis reported loose hairs, burrowing into otherwise normal tissue, inducing a foreign body reaction leading to secondary pits and cyst formation [14]. The source of the hair can either be the natal cleft itself in hirsute individuals, or hair from the head or back that falls into the natal cleft. The hair follicle becomes distended and obstructed leading to oedema and inflammation. Subsequently, a chronic abscess may develop, with a track draining it known as a sinus [12,15]. Furthermore, epidermal and deep tissue disruption are amplified by changes in the cleft microenvironment including increased moisture, anaerobic environment and bacteria in the natal cleft. Anaerobic bacteria (Bacteroides and Enterococci) predominate in the development of follicular infection and abscess formation and subsequent wound breakdown following surgery [16,17]. However, in 49 postoperative wound complications, aerobic bacteria was isolated in 43% of cases vs 40% anaerobic isolates [17]. Moreover, preoperative antibiotic usage did not shown reduction in the wound complication or recurrence rate after 30–42 months followup [17,18]. Therefore, the role of bacteria in initiating, persisting and recurrent pilonidal sinus disease evolves with disease progression and host response.

These factors have implications for both the extent of disease expression and progression. This was incorporated in a mathematic model following review of over 6000 patients [12]. The three primary variables were [1]: loose hair or ‘‘invader’’ (H) applies some [2] force (F), which is influenced by secondary factors such as the depth, narrowness, and friction of the natal cleft to create an insertion process. The third factor of vulnerability, (V), refers to the local tissue susceptibility. In this model, the primary sinuses represent the hair entry sites and secondary sinuses represent the exit points [19]:

Pilonidal Disease = Hair (H) × Force (F) × Vulnerability (V)^2^

The nature and variability of these causative elements have implications for persistent or recurrent disease. For example, the type and number of bacterial colonies present may be related to delayed wound healing following treatment [20]. Furthermore, deep tissue hypoxia is implicated in persistent pilonidal disease, with healing of complex wounds demonstrated by moving the suture lines to the open air [14]. These in turn have influenced various surgical treatment strategies.

## 3 Burden of disease

Pilonidal disease presents a significant disease burden worldwide affecting the working age population. The condition is more common in Caucasian males with a reported incidence of 1.1% [21]. In the United States, nearly 70,000 patients are diagnosed with this potentially morbid condition each year [5]. Figures from the UK Office of Population Censuses and Surveys recorded 7000 patients requiring inpatient treatment for pilonidal disease in England [22]. More recently, 13,329 hospital admissions for pilonidal disease were recorded in the UK NHS in 2012 [23]. In addition, a meta-analysis of 15 studies with minimum 5-years follow-up showed an overall recurrence rate of 13.8% following pilonidal surgery. This included 17.9%, 16.8% and 10% for open wounds, midline closure and off-midline closure respectively [24]. Furthermore, a Swedish study reported the cost of a conventional wide excision and closure for a pilonidal sinus to be EUR6222 per patient with a recurrence rate of 32% at 5 years [25]. Although healthcare costs are not widely reported, similar costs in other developed countries would imply a significant financial burden which would be higher still if the wound healed by secondary intention. Finally, the social impact on young people is also significant affecting interpersonal relationships, education and social activity [26]. The burden of disease thus has both quality of life and financial implications due to the high propensity for the disease to recur coupled with the likelihood that the patient may need more than a single treatment intervention along with high complications rates. The integrated care needed to achieve a good a clinical outcome often involves frequent community practice nurse input.

## 4 Operative management of pilonidal disease

The number and variety of published techniques testify to the complexity of treating pilonidal disease, with no single procedure superior in all respects. The most effective emergency management of a pilonidal abscess is simple incision and drainage [27]. However, surgical management of chronic and recurrent disease is more controversial. Numerous studies have been put forward advocating one excisional treatment over another, but many of these studies are weighed down by lack of control groups or short-term follow-up. Furthermore, pilonidal sinus excision with or without primary closure can be performed in different ways, either through a midline or lateral incision. In addition, recurrent pilonidal sinus disease after operative intervention presents a difficult challenge with long-term recurrence rates between 10 and 30% reported [24,28,29]. Recurrence can be divided into two groups: Early and late. Early recurrence is due to failure to identify one or more sinuses at operation, whereas late recurrence is usually due to secondary infection, residual hair or debris not removed at operation, inadequate wound care or insufficient attention to depilation [30]. The following section reviews the main methods for operative management.

Most procedures can be classified into one of four categories:

• Incision and drainage
• Excision and healing by secondary intention
• Excision and primary closure
• Excision with reconstructive flap techniques

## 5 Incision and drainage

There is no controversy surrounding the emergency approach for the treatment of acute disease with a simple incision and drainage required for an abscess [27]. This is a simple procedure that involves making an elliptical incision in the abscess just off the midline. The mouth of the wound should be sufficient to allow packing of the entire wound cavity. Curettage to remove dead or infected tissue in the wound improves the rate of healing, with 90% completely healed at one month, compared to just 58% healed at 10 weeks in the absence of curettage [20]. Healing by secondary intention has the advantage of allowing free drainage of infected material and debris although the patient will require regular wound care and the discomfort of packing until the wound has closed. In a retrospective study mean number of days off work following incision and drainage was 20 [27]. Furthermore, around 60% of patients treated in the acute setting do not require further surgical intervention after initial treatment. However, after complete healing about 10–15% will have abscess recurrence. In addition, following a single incision and drainage procedure, 40–60% will go on to develop a pilonidal sinus requiring further surgery. Pits or sinuses can be excised as part of the initial incision and drainage procedure, but these can be obscured by oedema and are often overlooked [14]. The recurrence rate can be reduced to 15% if a second procedure to excise pits and sinuses is performed after five to seven days [7].

## 6 Wide excision and healing by secondary intention

Wide excision of an elliptical wedge of skin and subcutaneous tissue down to the pre-sacral fascia is designed to remove all the inflamed tissue and debris allowing the wound to granulate from its base [16]. The excised dimensions should be of sufficient width at both the mouth and base of the wound to allow packing with ease. The base itself should be relatively flat and comparable in size to the mouth of the wound. A narrow V-shaped wound without a flat base is more difficult to pack and has the tendency to bridging and subsequent infection. The procedure necessitates general anaesthesia and hospital stay for a few days postoperatively. The principal advantage is low recurrence rate but the downside is a lengthy healing time (8–10 weeks) and high direct and indirect costs associated with inpatient care, follow-up wound care and days lost from work [31]. Despite this there is a role for wide excision in those with extensive chronic disease and following failed primary closure. Additionally, excisional techniques that minimize the wound can help reduce morbidity and healing time [32,33]. A study of 570 patients treated with minimal but complete excision under local anaesthetic showed a recurrence rate of less than 5% with mean followup of 4.7 years [32].

Furthermore, excision with marsupialization has been shown to have a better outcome. De-roofing of the tracts also minimizes the midline wound and shortens healing time. This approach is also effective in the presence of an abscess with recurrence reported to be less than 13% [19]. The technique involves opening the sinus tracts in the midline to include any secondary tracts. The posterior and lateral fibrous tissue is then left in situ and sutured to the wound base. The goal is to reduce the effective wound healing area thus reducing the healing time. Several studies have evaluated the effectiveness of incision with marsupialization when compared with excisional therapy. Solla and Rothenberger reported 150 patients in which 83% underwent marsupialization and had a recurrence rate of 6% [34]. Furthermore, Karakayali concluded that although healing time and postoperative care was longer in patients following marsupialization compared with excision followed by flap closure, other factors such as quality of life, return to work time, and pain scores favoured de-roofing and marsupialization [35].

For post-operative wound care following incision and drainage or excision procedures allowed to heal by secondary intention, the wound is packed with an alginate dressing. Following this, the wound can be managed with an appropriate secondary dressing often performed in the community setting. The dressing keeps the wound open preventing premature closure of the wound edges [36]. Several comparison studies have demonstrated this form of healing to take much longer compared to primary closure techniques [31] although minimizing the excised area can shorten the time-period for healing by secondary intention [33].

## 7 Excision and primary closure

Closure of the wound is more cosmetically acceptable and associated with a shorter healing time and reduced time off work compared to healing by secondary intention [13,37,38]. However, this benefit may be offset by potential higher risk of recurrence and wound infection [13]. In a prospective randomised trial failure of primary healing was significantly associated with early recurrence of disease [39]. In the same study the use of preoperative antibiotics did not influence the recurrence rate. Furthermore, when infection intervenes, the wound must be laid open and healing time is longer than if the wound had been treated by secondary intention initially. In addition, the scar can be sited over the midline or displaced laterally with one-year recurrence rates of 18% and 10% respectively [7]. Moreover, a systematic review of 6 studies indicated off-midline closure to be associated with faster healing times, reduced surgical site infection and reduced recurrence compared to midline wound closure [13] This was supported by a recent meta-analysis of 15 studies demonstrating 5-year recurrence of 10% for off-midline closure compared to 16.8% for midline closure [24]. Interestingly, healing by secondary intention had a higher recurrence rate of 17.9% indicating the need for long term followup in patients which may favour primary closure.

There are excisional procedures with technically more demanding forms of flap-reconstruction used for primary closure. Their use is generally restricted to recurrent or complex pilonidal disease in order to cover the defect with healthy tissue with a good blood supply following removal of diseased tissue [11]. Moreover, these procedures aim to flatten the natal cleft to reduce friction and reduce local warmth, moisture and hair accumulation. Firstly, Bascoms method (Cleft-Lift) uses incision, drainage and curettage through a lateral incision combined with excision of midline pits and a small amount of surrounding tissue [7]. A section of the cavity wall opposite the incision, lateral to the midline, is raised as a flap to close the defect. This is accomplished by suturing the flap to the underside of the skin bridge formed between the incision and the midline. In a study of 218 day surgery patients treated with Bascom's procedure, 6% developed a postoperative abscess requiring further drainage and 10% had recurrence requiring further surgery (mean follow up of 12.1 months (1–60 months)) [40]. Further studies have demonstrated greater than 90% short-medium term healing rates, including for recurrent disease [[41], [42], [43], [44]]. In addition, Karydakis pioneered raising a flap to overlap the midline with the scar sited to one side to reduce postoperative hair entry [14]. The fasiocutaneous flap is sutured to the sacrococcygeal fascia thereby reducing midline tension. A study of 6545 patients demonstrated a wound complication rate of 8% and recurrence rate of 2% [12]. Furthermore, the rhomboid/Limberg flap also excises the sinus tracts down to the pre-sacral fascia but utilizes a rotational flap to ensure coverage of the defect [45]. Whilst the technique also has low medium term recurrence rates, the technique may take longer to perform due to the larger area needed to mobilize compared to the Karydakis technique [[45], [46], [47]]. Finally, two further local advancement flaps can be used. The *Z*-plasty flap uses both skin and muscle to close the defect following excision [48]. In comparison to healing by secondary intention, there is no difference in complication rate with faster healing times over 22 months [49]. Moreover, the V–Y flap can be used unilaterally or bilaterally and serves to eliminate the gluteal cleft [11]. However, this requires a midline closure which is shown to have worse outcomes in systematic reviews [50] although good outcomes have been reported in very small case series with up to 5 years followup in both primary and recurrent disease [[51], [52], [53]]. Thus, a variety of complex primary closure techniques have shown good long-term outcomes in comparison to healing by secondary intention and when performed by appropriately trained surgeons. These techniques require general anaesthesia and depending upon complications, further medical input and/or hospital stay.

## 8 Non-operative treatment

Several non-surgical treatment strategies including phenol injection, fibrin glue, laser treatment, cryotherapy, VAC therapy and antibiotics have been reported [11,54,55]. Injecting 1–2 ml of 80% Phenol solution has been used to obliterate the epithelial lining of the sinus tracts either as primary or adjunctive therapy [56,57]. This is a closed technique under local anaesthetic whereby injection of phenol into a sinus causes sclerosis and gradual closure. Hegge et al. reported 3-year recurrence rates of 6.3% (95% CI 1.3%–17.2%) [58]. However, the patients required multiple treatments, in some cases up to 9 injections. In a further study of 41 patients, the majority of whom required 2–3 injections, reported a 95.1% success rate at 2 years followup [59]. The mean recovery time was 42 days but with reduced time off work. Moreover a randomised trial of 140 patients showed reduced healing time, operation time and pain scores with similar recurrence rates around 39 months in the phenol injection group vs excision with open healing group [60]. This trial was not blinded however, and the excision group had a higher incidence of previous abscesses which may have confounded results. An extensive review supportive of phenol treatment showed an overall success rate of 89% at 2 years although the authors noted a lack of high quality evidence [61]. The procedure is not time consuming but requires frequent repetition, has a high recurrence rate and there is a risk of cellulitis or abscess formation following administration. Additionally, other methods such as fibrin glue or antibiotics have poor evidence to support their use [61,62]. These factors have led to surgical techniques being generally favoured over non-surgical methods.

## 9 The future of pilonidal sinus surgery: the shift towards endoscopic treatment

The significant morbidity and recurrence of pilonidal sinus disease arising from traditional invasive surgical techniques has led to the development of less invasive, endoscopic methods for more targeted treatment of the disease. The aim of such treatments is to reduce the morbidity arising from excisional surgery whilst demonstrating efficacy in eliminating factors driving the disease process. Endoscopic pilonidal sinus treatment (EPSiT) was first described by Meinero et al., in 2014 [63] along with Video-Assisted Ablation of Pilonidal Sinus (VAAPS) reported by Milone et al., providing a magnified view of the tracts [64]. The technique is based on a diagnostic and interventional phase. A fistuloscope is inserted through a 0.5 cm circular incision once the external opening is excised. The initial phase identifies hair, debris and accessory tracts helping to plan for the interventional stage. Subsequently, hair and hair follicles are removed under direct vision using endoscopic forceps followed by electrocautery ablation of the granulation tissue lining the main and accessory tracts and finally removal of necrotic tissue. Thus, the basis of EPSiT/VAAPS is to target the hair invagination driving the pathogenesis of pilonidal sinus disease and prevent further growth in the tract, which heals by secondary intention. Moreover, it is performed using local anaesthetic and does not require regular packing change but does require wound review and compliance with wound hygiene instructions. In the initial series of 11 patients, no recurrences were reported in 6 months and patients returned to work between 1 and 5 days [63]. Furthermore at 1 year followup there was only 1 recurrence amongst 27 patients in a further case series which reported high levels of patient satisfaction [64]. In a randomised trial of 145 patients comparing VAAPS with Bascoms procedure showed reduced time off work, less pain and greater patient satisfaction allbeit recurrence at 1-year and post-operative complications were similar between the 2 groups [65]. Furthermore, 5-year followup of these patients revealed a similar long-term recurrence rate between VAAPS and Bascoms procedure, with VAAPS being more cost-effective and leading to better patient satisfaction [66]. An interesting variation of this technique was the endoscopic application of crystalized phenol, which showed no recurrence in 23 patients at 24 months [67]. Additionally, in a retrospective study of 80 patients comparing VAAPS to minimally invasive surgical techniques such as sinusectomy and primary closure, a significantly reduced recurrence rate at 5-years (7.5% vs 25%) was attributed to improved detection and clearance of all tracts in VAAPS [68]. There was no difference in pain scores, patient satisfaction or time off work between groups. Moreover, a systematic review compared 1RCT and 4 case series applying EPSiT to 5 RCTs reporting minimally invasive surgical techniques (sinusectomy, sinotomy, and trephining) covering 820 patients [69]. The complication rate and return to work time was similar between EPSiT and other minimally invasive surgical technique but superior to traditional surgical techniques. In addition, several recent systematic reviews have analysed EPSiT/VAAPS application from published case series along with the one randomised trial published to date. Tien et al. showed patients undergoing EPSiT/VAAPS to have reduced time off work, low short term recurrence and high satisfaction scores although the technique took longer to perform and required specialist, expensive equipment [70]. Furthermore, Emile et al. assessed 497 patients across 9 studies (6 prospective, 2 retrospective and 1 RCT) [71]. They reported a treatment failure rate of 8%, recurrence rate of 4%, complication rate of 1.1%, mean return to work in 2.9 days and mean time to healing of 32.9 days. EPSiT/VAAPS is therefore a safe surgical technique that has shown similar short-term efficacy to other minimally invasive surgical techniques in managing pilonidal sinus disease at least in the short term. As expected, morbidity from endoscopic treatment is far less than conventional open surgical techniques and the small sized wound leads to a better cosmetic outcome [72]. Challenges remain with this new technology, including standardization of technique to reduce heterogeneity in systematic reviews, and obtaining high quality prospective trial data that reduces the inherent selection bias in the current retrospective dataset. Moreover, the costs in setting up an endoscopic approach and learning curve for surgeons in a procedure that takes longer to perform at present along with the possible need for repeat treatments needs to be factored.

## 10 Conclusion

Pilonidal disease is a complex condition to treat that causes both discomfort and embarrassment to sufferers with high direct costs to the healthcare system and indirect costs through absence from work. Incision and drainage with curettage is recommended for treating pilonidal abscesses. Wide excision and either healing by secondary intention or primary closure is the commonly applied treatment for most chronic pilonidal sinus disease at present. No one treatment modality has gained universal acceptance and recurrent disease with the attendant morbidity remains a challenge. Thus, the surgical management of complex or recurrent pilonidal sinus disease should be under a surgeon with an interest in this condition and based on up-to-date evidence. Regardless of the surgical technique applied, standard principles of wound care are essential and patient education plays a critical role in this.

Off-midline primary closure with the various flap-techniques have shown improved patient outcomes and should become more accepted amongst surgeons. However, controversy exists about the benefit of primary closure with conflicting evidence supporting this approach. Moreover, these complex procedures can be performed in the daycase setting and along with reduced risk of complications or recurrence compared with midline closure, lead to less absence from work and increased cost-effectiveness. However, over-treatment with a wide excision and reconstructive flap operation for relatively simple pilonidal sinus disease can prolong hospital stay, increase time-to-work and lead to unacceptable cosmetic outcomes. Although the majority of recurrences occur in the first 5-year period post-surgery recurrences have been reported later. Therefore, to improve the body of evidence, future prospective randomised trials must ensure adequate long-term followup and report on patients requiring multiple treatments which is a key quality indicator in managing pilonidal sinus disease.

The shift towards endoscopic therapy may lead to reduced surgical morbidity although current evidence is limited in support of this. The downside includes the potential costs of equipment and the inevitable learning curve that will be required. Further challenges include the very limited evidence base centred on a small number of trials many of which are subject to bias. Moreover, patient selection will be key as endoscopic therapy will provide most benefit if treatment can be achieved through a single opening. Several patients may still require either minimally invasive surgery or wide excision, but the aim will be to reduce this number with endoscopic treatment.

## Ethical approval

No ethical approval sought or required.

## Sources of funding

No funding from any source utilized in the completion of this project.

## Author contribution

Fahad Mahmood – Writing paper, drafting article.

Anwar Hussain – Data interpretation, Study Concept.

Akinfemi Akingboye – Study Concept, Approval of final manuscript.

## Registration of research studies

1 Name of the registry: N/A
2 Unique Identifying number or registration ID: N/A
3 Hyperlink to your specific registration (must be publicly accessible and will be checked): N/A

## Guarantor

Akinfemi Akingboye.

## Provenance and peer review

Not commissioned, externally peer reviewed.

## Declaration of competing interest

Nothing to declare.
